# Speed-Dependent Turning Strategies in Quadrupedal Locomotion: Insights from Computational Modeling

**DOI:** 10.64898/2026.01.12.699101

**Published:** 2026-01-12

**Authors:** Yaroslav I. Molkov, Mohammed A. Y. Mohammed, Tommy Stell, Amelia Harralson, Russell Jeter, Ilya A. Rybak

**Affiliations:** 1 Department of Mathematics and Statistics, Georgia State University, Atlanta, GA, USA; 2 Neuroscience Institute, Georgia State University, Atlanta, GA, USA; 3 Lehigh University, Bethlehem, PA, USA; 4 Department of Neurobiology and Anatomy, Drexel University College of Medicine, Philadelphia, PA, USA

## Abstract

Quadrupedal animals like mice navigate their environments through complex coordination of neural signals and biomechanical movements, enabling stable and directed locomotion. While many computational models simplify this process by assuming left-right symmetrical body movements and focusing on straight-line paths, real animals rely heavily on asymmetrical body movements to execute turns and adjust speed effectively. This study builds upon a previously developed model of quadrupedal locomotion proposed by (Molkov et al., 2024) in which forward movement of the body was driven by central neural interactions, biomechanics, and proprioceptive feedback. We extended this model to comparatively investigate possible mechanisms of steering by introducing three distinct asymmetrical strategies–body bending, lateral force application, and lateral limb shifting as well as their combinations–to explore their potential involvement in turning performance. By simulating these strategies across a walking speed range, we measured and compared their impact on turning curvature (the sharpness of the turn) and limb coordination. The latter was quantified through ratios of duty factors representing the relative time that a limb spent in contact with the ground compared to its counterpart on the opposite side. Our findings reveal that each strategy excels at different speeds: body bending allows sharp turns at low speeds, lateral force is most effective at medium speeds, and lateral shifting performs best at higher speeds. Our results suggest that animals select or combine turning strategies based on their locomotor speed or adjust speed to use a specific strategy. We also show that the forelimbs consistently play a primary role in steering, while the hindlimbs adjust propulsion and stability in ways that depend on the specific turning strategy. These results provide valuable insights into how spinal circuits and mechanical asymmetries work together to produce flexible, adaptive movement patterns, offering a robust framework for understanding locomotion in both biological organisms and robotic systems designed to mimic such behaviors.

## Introduction

Quadrupedal locomotion is characterized by a remarkable coordination of speed and limb movements, allowing animals like mice to adaptively change direction and navigate in complex and varied environments. This ability relies on the interplay between the central nervous system, which generates and modulates movement commands, and the musculoskeletal system that translates these commands into physical motion (Orlovskiĭ, 1999; Bertram, 2016). While straight-line locomotion has been extensively studied, the mechanisms governing turning–a critical maneuver that requires breaking bilateral symmetry–are less well understood. Many computational models simplify quadrupedal locomotion by assuming a symmetrical body and focusing on forward movement, often using Central Pattern Generators (CPGs) to produce rhythmic limb patterns (Danner et al., 2016, 2017). However, these simplifications fail to capture the dynamic asymmetries that real animals employ during turning, such as adjusting body posture or limb forces to change direction (Pycock, 1980; Walter, 2003; Gruntman et al., 2007; Cregg et al., 2020; Usseglio et al., 2020; Haagensen et al., 2022).

Turning is essential for survival, enabling animals to avoid obstacles, pursue prey, or evade predators. Unlike straight-line movement, turning demands the generation of asymmetrical forces and movements, which arise from intricate left-right asymmetric interactions between neural commands and biomechanical structures (Cregg et al., 2020; Usseglio et al., 2020). For instance, mice may bend their spines or reposition their limbs to execute sharp turns, strategies that are actively controlled rather than mere deviations from symmetry (Walter, 2003; Gruntman et al., 2007). Understanding these asymmetrical strategies is crucial for developing computational models that more accurately reflect biological locomotion and for informing the design of agile robotic systems capable of navigating complex terrains (Wei et al., 2018; Lee et al., 2020).

This study addresses this research gap by extending a simplified model of quadrupedal locomotion, originally developed by (Molkov et al., 2024), and using the extended model to investigate turning behaviors. The original model simulated straight-line walking in mice using sensory feedback to the CPG-based central neural controller to generate limb movements. Turnings are based on different biomechanical asymmetries. We introduced three controlled asymmetries–lateral body bending, lateral force application, and lateral limb displacement–to simulate turning and quantify the speed-dependent effects of each strategy and their combinations on sharpness of turning. Specifically, we measured curvature, which indicates the sharpness of a turn and characterized the dependence of the maximal achievable curvature on speed and the choice of a turning strategy. To investigate the related changes in limb coordination during turning, we analyzed duty factor ratios, defined as the ratio of the proportion of time a limb spends in contact with the ground (stance phase) relative to its total stride cycle for the left versus right limbs. Our objectives were to determine how these basic turning strategies influence turning performance at different speeds and to explore the distinct roles of forelimbs and hindlimbs in achieving directional control. By doing so, we aim to elucidate the interplay between spinal circuits and mechanical asymmetries, providing insights applicable to both biological locomotion and robotic system design. The findings offer insights into the neuromechanical mechanisms that quadrupedal animals employ for steering and navigation while moving.

## Model Description

The computational model used in this study is grounded in biomechanical principles observed in animal locomotion, emphasizing the role of proprioceptive feedback in control of locomotion (Pfeifer et al., 2007; Frigon et al., 2021; Molkov et al., 2024; Rybak et al., 2024). Unlike models that rely on pre-programmed neural oscillators, this model posits that complex rhythmic movements emerge from distributed sensory feedback loops and the intrinsic mechanical properties of the body (Full & Koditschek, 1999; Holmes et al., 2006); a concept also known as “morphological computation” (Owaki et al., 2013; Müller & Hoffmann, 2017).

### Model of the body

**Figure 1.**
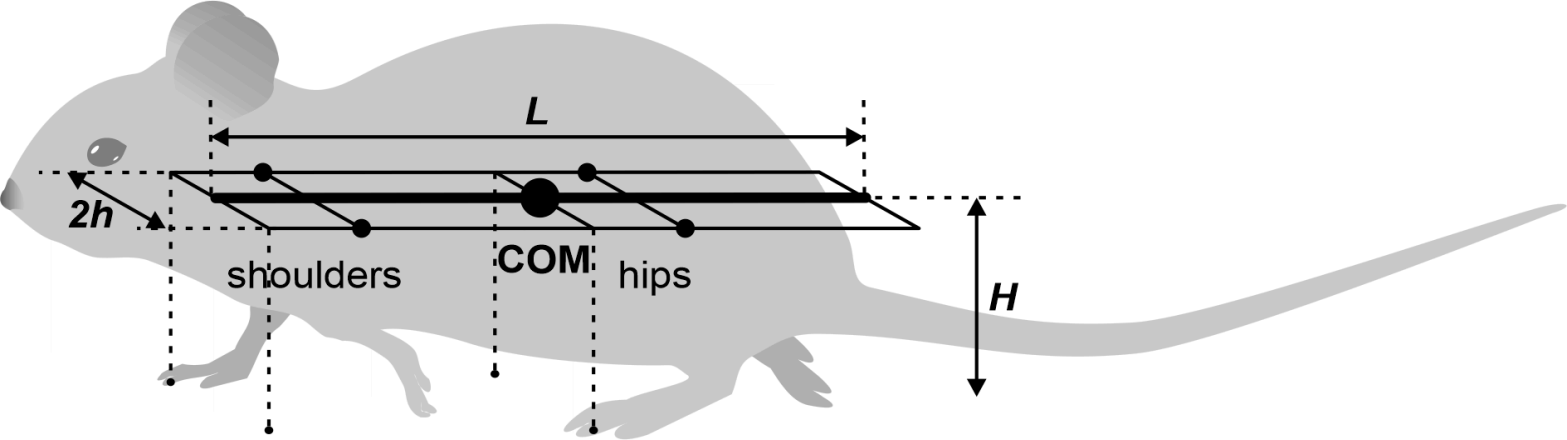
The mouse’s body as represented in the model.

In our model, the mouse body is represented as a rigid rod of length L possessing uniform linear density, oriented parallel to the ground at distance H (height of the mouse), and having three degrees of freedom: horizontal position (x, y coordinates) and orientation (Fig. 1). In the horizontal plane, the left and right “shoulder” and “hip” joints are equidistant, at a distance h from the rod, forming the body frame, a L×2h area rectangle. The center of mass (COM) is located in the middle of the body.

The body’s weight is supported by its limbs during stance. The initial paw positions are defined by the ground projections of the corners of the body frame as indicated by the vertical dashed lines in Fig. 1: the front of the frame for the forelimbs and the center (aligned with the COM) for the hindlimbs (Fig. 1). These positions, which are laterally displaced by h from the centerline, also serve as the default target positions for the corresponding paws during the swing phase. The maximum possible limb displacement in the horizontal plane from this initial position is D=L/2.

### Central controller and control of movement

The model has a central controller or Central Pattern Generator (CPG) consisting of four Rhythm Generators (RGs) each controlling one limb and operating as a state machine with two states defining the stance (ground contact) and swing (off-ground movement) phases of the corresponding limb. Sensory feedback signals that characterize limb loading, limb extension, and overall postural stability modulate these generators (by controlling the timing of stance-to-swing and swing-to-stance transitions) enabling adaptive locomotion (for details see Methods and (Molkov et al., 2024)).

Movement in the model arises from the interaction between a central neural controller and the mechanical components of the system. Each limb alternates between stance, where it contacts the ground to generate propulsion, and swing, where it moves freely to prepare for the next step. Load-sensitive receptors detect when a limb is bearing weight or unloading, directly influencing the timing of phase transitions and ensuring balance and forward motion.

During the swing phase, limbs do not contribute to propulsion. Limb loading is the vertical component of the ground reaction force which is calculated from the COM coordinates relative to limb positions. A limb transitions from the stance to swing phase when its loading becomes zero or negative, or its extension limit is surpassed. Swing-to-stance transitions are governed by a feedback control mechanism to prevent loss of balance (see (Molkov et al., 2024) and Methods).

The model’s equations of motion describe the dynamics of the mouse body in a horizontal plane, treating it as a rigid system with a center of mass (COM). These equations are derived from Newton’s second law applied to the planar motion of the COM and to the angular momentum for the body’s rotation around the COM. They incorporate kinematic friction to account for energy dissipation during locomotion. The primary forces acting on the system include propulsion forces generated by the limbs in contact with the ground and an inverted pendulum force that arises specifically during phases when only two limbs are supporting the body.

Ground reaction forces, which represent the interaction between the paws and the ground, are decomposed into **horizontal** and **vertical** components. The horizontal components drive forward movement and rotation, while the vertical components support the body’s weight.

### Horizontal forces and yaw torques

In the horizontal plane, each limb in stance generates a propulsion force of equal magnitude for all supporting limbs, directed along the paw’s displacement from its initial position during stance in the body’s coordinate system. This force acts as a key control parameter that influences the overall locomotor speed. To model energy losses from various unrepresented factors like muscle inefficiencies or ground interactions, a viscous friction force is included, which opposes the COM’s velocity and scales linearly with it.

When exactly two limbs are on the ground, gravity influences horizontal motion because the body cannot be fully supported vertically. In this scenario, the system behaves like an inverted pendulum, pivoted along the line connecting the two supporting paws. The COM’s projection deviates from this support line, creating a horizontal force component that pulls the COM away from the line, accelerating it perpendicularly to the line of support. If more than two limbs are supporting the body, the COM remains within the polygon formed by the paw positions, and no such inverted pendulum force is present. For rotational dynamics, propulsion forces create yaw torques because they are offset from the body’s COM.

### Vertical forces

The vertical components of the ground reaction forces, referred to as “limb loads” or “weight-bearing forces,” support the body’s weight and are computed based on the number of limbs in stance.

For support by more than two limbs, the total vertical force must equal the gravitational force on the body to prevent vertical movement. Additionally, the torques from these forces about the COM must balance to avoid pitch or roll, consistent with the assumption that the body frame remains in a horizontal plane. This setup forms a system of balance conditions: force equilibrium and two torque equilibrium equations (for the two horizontal directions). For three-limb support, this yields a unique distribution of loads. For four-limb support, an extra condition is imposed to distribute the load as evenly as possible. Loads must be positive for limbs to remain on the ground; if a load becomes zero or negative, it indicates unloading and triggers a transition to swing. Geometrically, positive loads require the COM to lie inside the support polygon formed by the paws which is a prerequisite of static stability.

For two-limb support, full weight balance is generally impossible unless the COM is exactly above the support line, and the total vertical force is reduced. Load distribution between the two limbs assumes no pitch along the support line, so the torques about the COM’s projection onto that line balance. This provides the individual loads for the supporting limbs. Crucially, during this phase, the total vertical load is not constant but varies dynamically. As the body moves under the influence of gravity (acting as an inverted pendulum), the COM inevitably moves away from the vertical equilibrium position, causing the total vertical ground reaction force to decrease. A negative time derivative of this total load (i.e., when the load is reducing) serves as a robust physical indicator of impending instability. In our model, we utilize this specific event—the instant the total load begins to decrease—as the “loss of balance” signal. This sensory feedback immediately triggers the swing-to-stance transition of one of the swinging limbs, thereby expanding the support polygon and restoring stability before a fall can occur.

For full mathematical details, including derivations and parameter justifications, see Appendix: Methods. In its baseline configuration, without intentional asymmetries, the model produces stable, symmetrical gaits that result in straight-line locomotion, consistent with typical walking patterns in mice (Molkov et al., 2024).

### Asymmetries for turning

**Figure 2.**
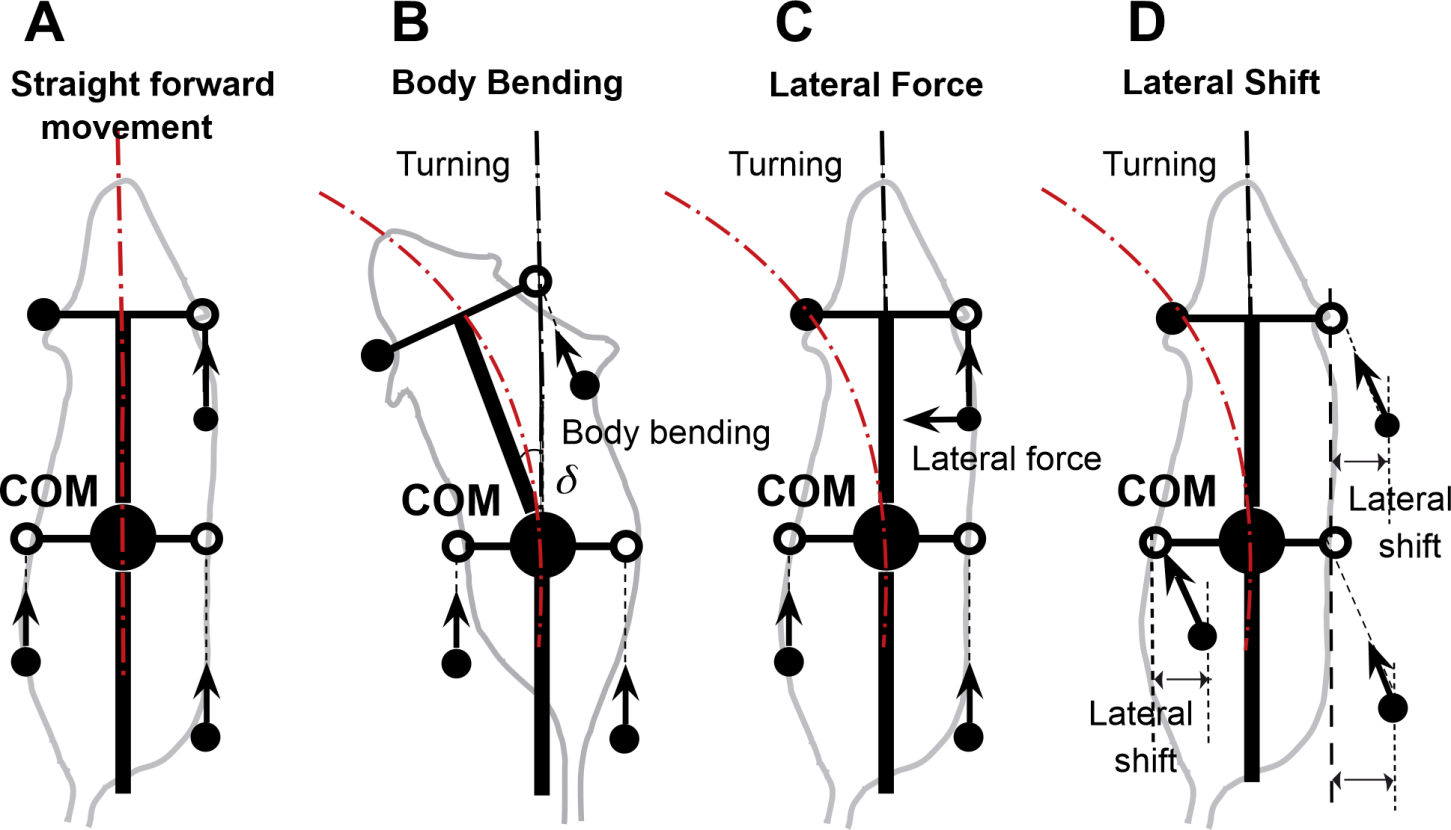
Straight forward movement (A) and three turning strategies based on: Body Bending (B); Lateral Force (C), and Lateral Shift (D). **(A)** In the baseline configuration, the model produces a stable, symmetrical gait resulting in straight-line locomotion. **(B) Body bending** introduces asymmetry through the non-parallel alignment of the hip and shoulder axes (simulating axial flexion), which shifts the center of mass inward and redistributes propulsive forces to facilitate turning. **(C)** The **Lateral Force** strategy involves applying a force perpendicular to the body axis, specifically at the forelimbs, to directly generate centripetal acceleration and active steering. **(D) Lateral Shift** is achieved by displacing the limb landing positions side-to-side relative to the body axis during the swing phase. This alters the base of support and effective lever arms for ground reaction forces, inducing lateral acceleration and rotation. Black filled circles represent the positions of limbs that are currently in stance phase (ground contact). Open circles indicate default target positions for the limbs during the swing phase. Arrows indicate the directions of propulsive forces.

To investigate turning, we extended this model by introducing three distinct forms of controlled asymmetry, designed to mimic strategies observed in real quadrupeds (Fig. 2). First, **Body Bending** allows the hips and shoulders to deviate from parallel alignment, simulating spinal flexion that reorients the body’s axis. Second, **Lateral Force** applies forces perpendicular to the body axis, primarily through the forelimbs, to induce rotational moments. Third, **Lateral Shift** offsets the side-to-side placement of limbs relative to the body’s midline, altering the distribution of ground reaction forces. These modifications enable the model to break from symmetrical gaits and generate turning behaviors, providing a platform to study how asymmetries influence locomotor control.

To explore turning behaviors, we systematically applied each asymmetrical strategy—body bending (Fig. 2B), lateral force (Fig. 2C), and lateral shift (Fig.2D) — to the computational model. This model extends the original proprioceptive feedback-driven framework for quadrupedal locomotion described in (Molkov et al., 2024). In the original model, the propulsion force generated by each limb during stance was directed strictly along the body’s longitudinal axis, assuming symmetrical and forward-oriented paw displacements. In this extended model, however, the propulsion force is instead directed along the displacement of the paw relative to its unperturbed initial position during stance in the body coordinate system. This modification allows for more flexible force vectors that can incorporate lateral components in asymmetric conditions, better capturing turning dynamics. All propulsion forces remain equal in magnitude across limbs but vary in direction based on actual paw movement, which emerges from the interplay of neural feedback and mechanical constraints. In particular, with the lateral shift asymmetry (described below), this redirection creates inherent lateral forces, as the shifted paw positions result in displacements that are not purely axial, thereby generating perpendicular components that create lateral acceleration and contribute to rotational torques without explicit lateral force addition.

Specifically, the asymmetries were introduced as follows:

#### Body bending (Fig. 2B):

This strategy simulates spinal flexion by introducing an angular deviation δ between the front (shoulder) and rear (hip) segments of the body. In the base model, the body is a single rigid rod with fixed parallel alignment of hips and shoulders. This alters the relative orientations of the limb attachment points: forelimb positions (ground projections at the front of the frame) and hindlimb positions (at the center) are recalculated in the body’s coordinate system, shifting the target paw positions during swing and the support polygon during stance. Consequently, this reorients the overall body axis over time, generating a rotational torque through asymmetric ground reaction forces without changing force magnitudes. The modified paw displacements under body bending influence the propulsion force directions, potentially introducing slight lateral components.

#### Lateral force (Fig. 2C):

This involves adding a perpendicular component to the propulsion forces, which in the base model are determined by paw displacements but effectively axial in symmetric gaits. The lateral force was applied perpendicular to the body axis, directed inward toward the turn (e.g., leftward for a left turn). This was targeted at the forelimbs to mimic steering. Hindlimbs received zero lateral component to emphasize forelimb-driven rotation. This addition affects the horizontal equations of motion by introducing asymmetric yaw torques (computed as the cross product of the force and the vector from COM to paw position), leading to rotational acceleration while preserving total forward propulsion.

#### Lateral shift (Fig. 2D):

This kinematic asymmetry adjusts the side-to-side limb placements relative to the body’s midline. In the base model, left and right limbs are symmetrically displaced by h (half-width) from the centerline. To implement the shift, an offset s was applied asymmetrically: for a left turn, left-side limbs (fore and hind) were shifted inward by s (reducing their distance from midline to h−s), while right-side limbs were shifted outward to h+s. This modifies the initial stance positions and swing target positions in the body’s coordinate system, altering the geometry of the support polygon formed by the paws. As a result, the distribution of vertical loads and horizontal ground reaction forces becomes biased, shifting the COM’s effective path and inducing curvature through changes in the inverted pendulum force (during two-limb support) and overall torque balance. Notably, because propulsion forces follow paw displacements, the asymmetric positions naturally produce lateral force components, enhancing turning without explicit perpendicular additions.

### Axial (sagittal) shift of paw placement as a stride control mechanism

**Figure 3.**
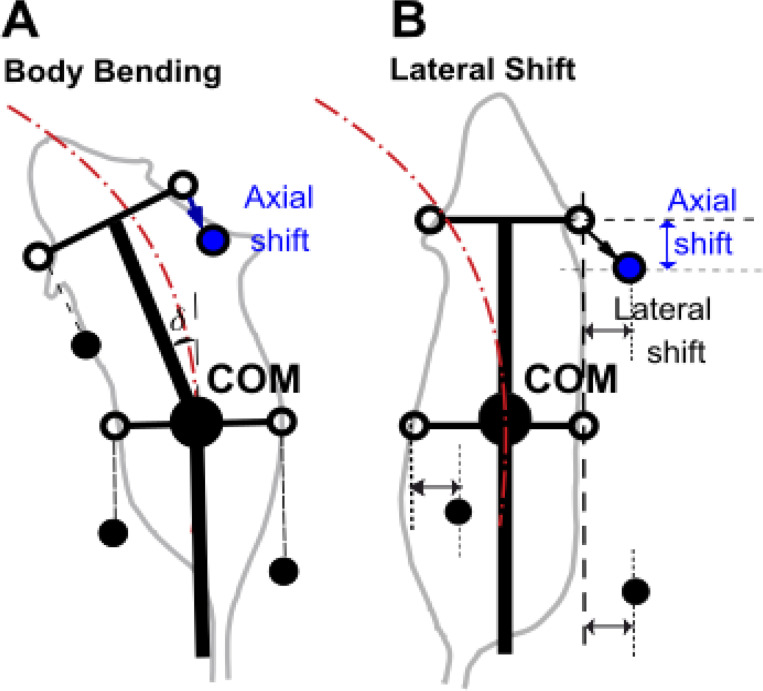
Axial shift implementation in turning strategies. **(A)** Body bending strategy. **(B)** Lateral shift strategy. Black filled circles represent the positions of limbs that are currently in stance phase (ground contact). Open circles indicate default target positions for the limb during the swing phase. Blue circles indicate the adjusted target position in the presence of a non-zero axial shift. The arrows indicate the displacement from the default target (open circle) to the shifted target (blue circle). This posterior shift adjusts the foot placement relative to the body’s center, modifying the support polygon during the turn.

To counteract the destabilizing effects of turning asymmetries, we implemented a stride control mechanism defined as an axial (or sagittal) shift of paw placement during the swing-to-stance transition (Fig. 3). In the baseline model, limbs are targeted to land at a fixed anterior position relative to the body frame. However, during curved locomotion, maintaining this default anterior placement can lead to the center of mass (COM) drifting outside the support polygon, particularly when the body axis is rotated relative to the velocity vector.

The axial shift mechanism introduces a rearward adjustment to the target footfall position. By shifting the paw placement caudally (backward) along the body’s longitudinal axis at the moment of touchdown, the effective stride length is shortened. This adjustment has two critical effects on static stability:

1. **Support Polygon Centering:** Shifting the touchdown position backward effectively moves the support polygon posteriorly relative to the COM. This ensures that the COM remains securely within the boundaries of the support polygon for a greater proportion of the gait cycle, preventing the “roll-over” instability observed in the absence of stride control.
2. **Gait Frequency Modulation:** By shortening the stride length while maintaining propulsion velocity, the axial shift implicitly increases the stepping frequency. This allows for more frequent updates to the support polygon, reducing the duration of the unstable two-leg support phases and providing more continuous static stability during the turn.

## Results

Our primary objective was to comparatively investigate the three turning strategies described above, focusing on the maximal achievable turning curvature during stable locomotion and its dependence on velocity. While a certain degree of left-right asymmetry is necessary for turning, each type of asymmetry inherently destabilizes locomotion, imposing specific limitations on achievable curvature and speed.

To address this, we first evaluated the performance of these strategies without stride control. We then compared these results to simulations that included stride control (implemented via an axial shift of the limb’s footfall target position). As we show, the addition of stride control significantly expands the range of velocities and curvatures available for stable locomotion. Our analysis of curvature as a function of velocity revealed distinct performance profiles for each strategy, delineating regions of instability where the model could not maintain stable locomotion. These regions represent physical constraints similar to those faced by biological organisms, where excessive speed or asymmetry can lead to a loss of balance or necessitate gait transitions (Lee et al., 2020).

### Asymmetries destabilize walking without stride control (no axial shift)

**Figure 4.**
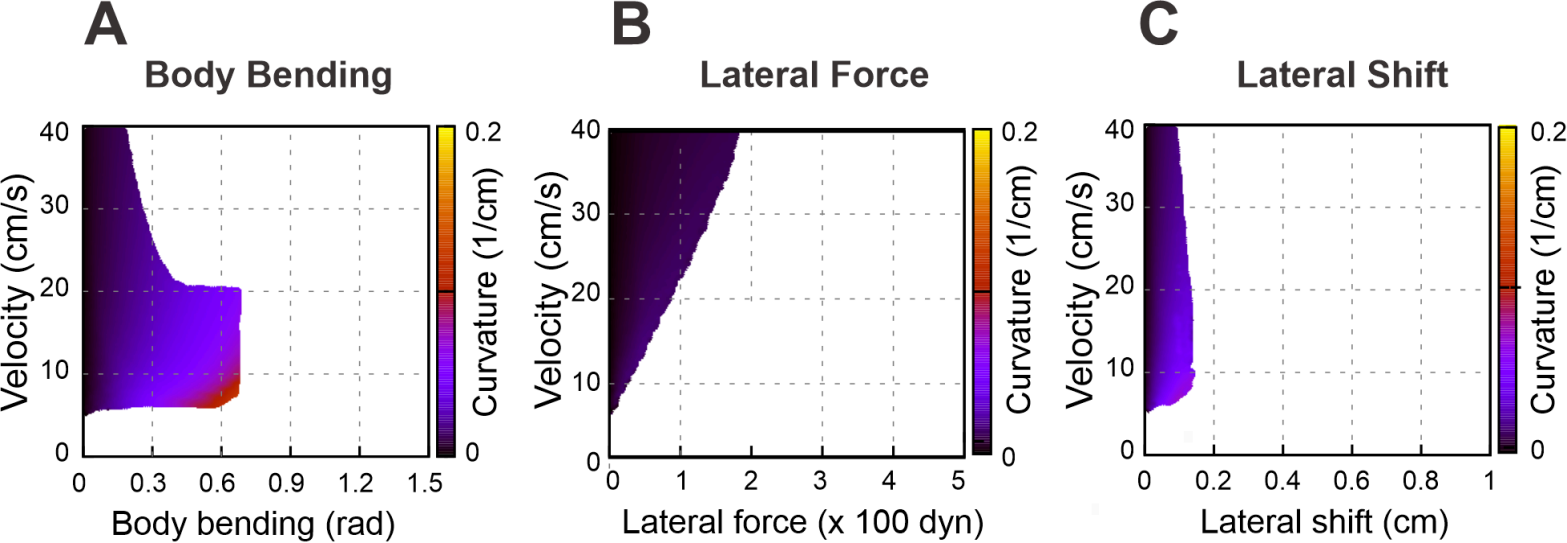
Heatmaps illustrating the turning curvature for three distinct strategies: body bending (A), lateral force (B), and lateral shift (C). These heatmaps show the relationship between turning curvature, velocity (regulated by the propulsion force), and the intensity of each strategy. Shaded areas denote stable walking, while white regions indicate instability, signifying that the model was unable to sustain stable locomotion.

To explore the relationship between turning curvature and locomotor velocity for different turning strategies, we constructed corresponding heatmaps for each: body bending (Fig. 4A), lateral force (Fig. 4B), and lateral shift (Fig. 4C). In these maps, successful turns are represented by the shaded areas, whereas the white areas indicate instability and therefore delineate the system’s dynamic boundaries. A key observation is that none of the turning strategies yield high curvatures when the limbs are set to their default position at the swing-to-stance transition.

White areas in Fig. 4A-C indicates unstable locomotion. When an asymmetry is applied (body bending, an added lateral force, or a lateral limb shift), an unbalanced torque about the body COM (yaw) is produced. That torque rotates the body frame, changing the ground projections of the paw attachment points relative to the COM. Because propulsion forces act through those paw positions, rotation tends to reinforce the yaw (a positive feedback loop) which can lead to misalignment of the body axis and the COM movement direction. Stability in our model is geometric: the vertical projection of the COM must remain inside the convex polygon whose vertices are the paws in contact with the ground. As asymmetry grows and the body rotates, the COM projection moves laterally toward the outer side of the turn and the COM projection may cross the support boundary of the outer limbs. Once the COM moves beyond that outer support line the model can no longer produce positive limb loads that keep the body upright — vertical loads go to zero or negative on inner limbs, the remaining outer limbs cannot generate counter-torque, and the body rolls over.

### Axial (sagittal) shift at swing-to-stance transition enhances efficiency across all three strategies

**Figure 5.**
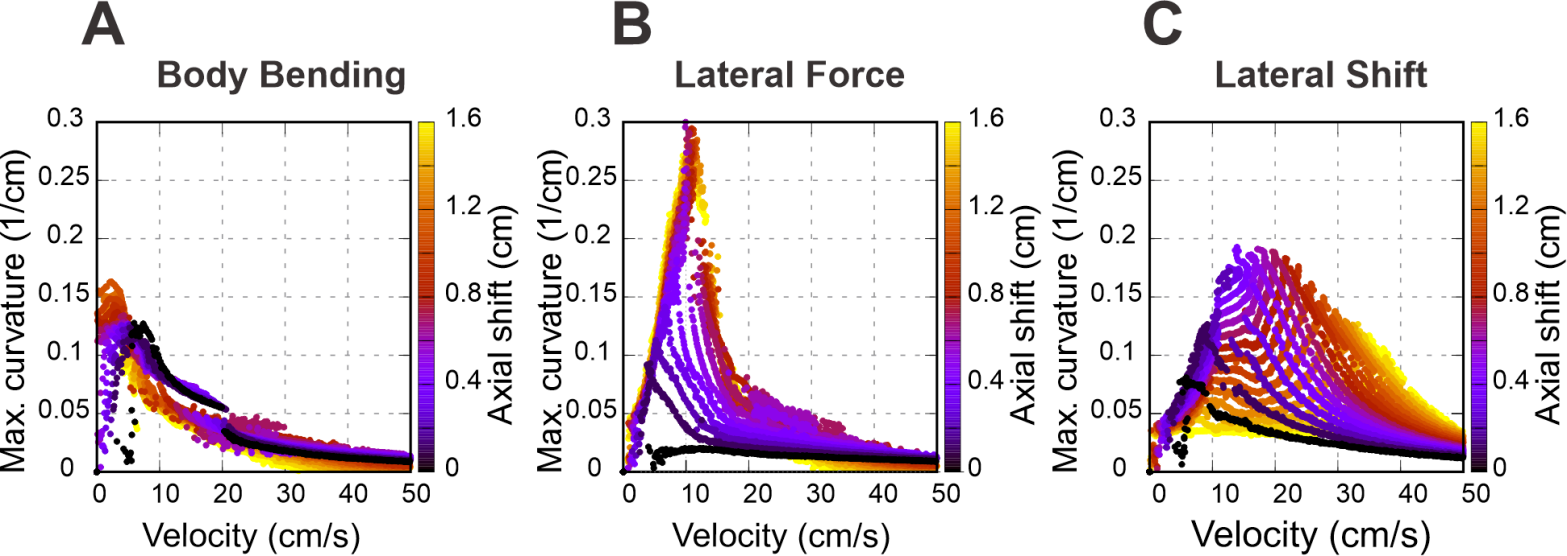
Speed-dependent effects of axial shift on turning performance. Maximum achievable curvature plotted against velocity for body bending (A), lateral force (B), and lateral shift (C). Color corresponds to the magnitude of the axial shift in centimeters. Note that body bending only allows for sharp turns at low speeds and lateral force achieves the highest overall curvatures at medium speeds; both saturate at ≈1 cm shift. In contrast, in the lateral shift strategy, the axial shift pushes the performance curve toward higher velocities, peaking at a smaller axial shift of ≈0.4 cm.

**Figure 6.**
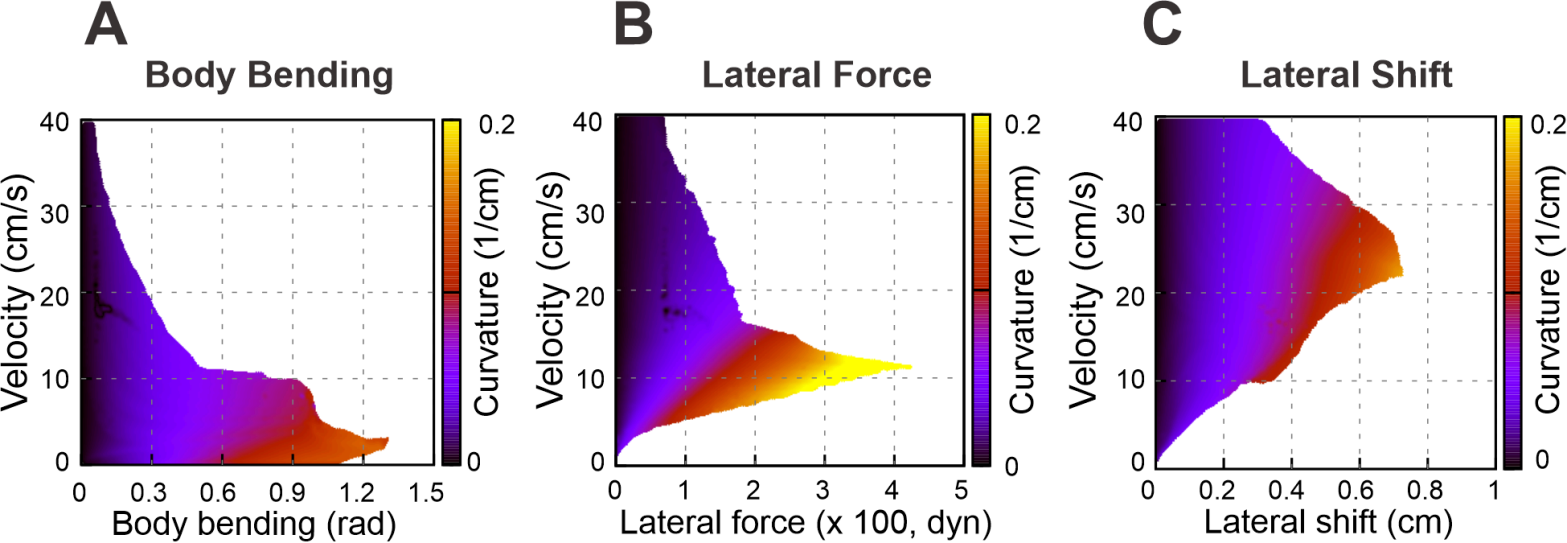
Stability heat maps showing the feasible turning regions in the parameter space of velocity vs. strategy intensity. These maps represent the “optimal” axial shift conditions: 1 cm for body bending (**A**), 1 cm for lateral force (**B**), and 0.4 cm for lateral shift (**C**). Compare to the zero-shift conditions in Fig. 4A-C.

To expand the range of stable locomotion velocities and turning capabilities we implemented the stride control via an axial (sagittal) shift—a posterior adjustment of the limb’s footfall target position. While this mechanism generally improved stability, the nature of the enhancement varied distinctly depending on the turning strategy employed. These effects are detailed below and illustrated in Figures 5 and 6.

Specifically, Fig. 5 demonstrates how the maximal turning curvature changes with the velocity at different values of axial shift for each turning strategy considered: body bending (Fig. 5A), lateral force (Fig. 5B), and lateral shift (Fig. 5C). Figure 6A-C shows the heat maps for each turning strategy indicating the feasible turning regions in the parameter space of velocity vs. strategy intensity at “optimal” axial shift conditions. The latter can be compared with the corresponding maps in Fig. 4A-C calculated with zero axial shifts.

The most pronounced effect of the axial shift on the body bending strategy (Fig. 6A) is the stabilization of turns at low velocities (<5 cm/s). Without this shift, body bending is ineffective at slow speeds due to instability (Fig. 4A). The axial shift counteracts this, allowing the strategy to function effectively even at very low speeds. The performance benefits for this strategy increase with the magnitude of the shift up to a saturation point of approximately 1 cm.

For the lateral force strategy (Fig, 6B), the axial shift facilitates turning primarily at medium speeds (5 – 15 cm/s). With the axial shift applied, this strategy is capable of generating the highest maximum curvatures of all three methods. Similar to body bending, the efficiency gains for lateral force saturate at an axial shift of approximately **1 cm**, beyond which no further improvement in curvature is observed.

The effect of axial shift on the lateral shift strategy (Fig. 6C) differs qualitatively from the others. Instead of simply extending the range, the axial shift displaces the performance curve, shifting the optimal turning capability from intermediate speeds to higher speeds (15 – 40 cm/s).

Furthermore, the magnitude of axial shift required to peak performance is lower; the turning curvature maximizes at approximately 0.4 cm of axial shift, distinguishing it from the 1 cm saturation point observed in the other two strategies.

**Figure 7.**
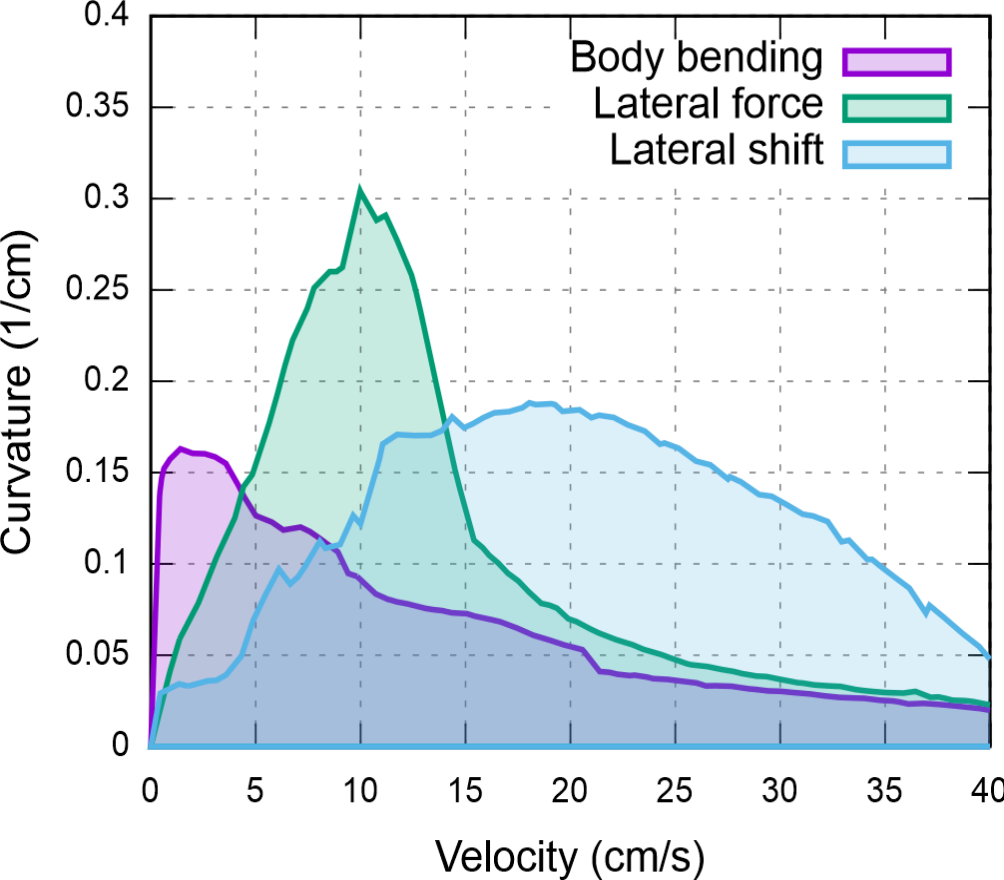
Comparison of maximal turning performance across strategies. Each curve represents the maximum achievable curvature as a function of locomotor velocity for the three primary turning strategies: body bending (purple), lateral force (green), and lateral limb shift (blue). The corresponding shaded regions describe the stable performance envelope under axial shift conditions optimized for each speed. The results reveal a clear speed-dependency: body bending excels at low speeds (<5 cm/s), lateral force generates the highest absolute curvatures at intermediate speeds (5–15 cm/s), and lateral shift maintains superior stability and maneuverability at higher velocities (>15 cm/s).

The comparative analysis summarized in Fig. 7 demonstrates that no single strategy is universally optimal; instead, the effectiveness of a turning mechanism is fundamentally tied to the animal’s locomotion speed. At low velocities, the body bending strategy leverages static stability to achieve sharp turns through axial flexion. As velocity increases, the dynamic requirements of steering favor the lateral force strategy, which produces the greatest turning sharpness by utilizing forelimb-driven torques. Most notably, the lateral shift strategy becomes the most effective at high speeds, where kinematic adjustments to the base of support are necessary to prevent roll-over instability.

This suggests that animals may select strategies based on locomotion speed, as seen in robotic models (Wei et al., 2018).

### Combining strategies

In previous sections, we focused on the analysis on three distinct turning strategies. At the same time animals, particularly small quadrupeds like rodents, often combine multiple strategies to perform desired turning under different conditions (Pycock, 1980; Walter, 2003; Gruntman et al., 2007; Usseglio et al., 2020; Haagensen et al., 2022). To investigate whether a combination of different strategies leading to multiple asymmetries could extend the range of achievable curvatures or bridge the performance gaps between individual strategies, we performed brute-force optimization on pairs of strategies. We defined the maximum magnitudes for each mechanism based on the upper limits used in our previous single-strategy heatmaps: 2 rad for body bending, 500 dyn for lateral force, and 1 cm for lateral shift.

For each pair of strategies, we explored a continuum of combinations defined by a mixing parameter 𝑠 (ranging from 0 to 1). For each 𝑠 we simultaneously activated two distinct control parameters such that the first strategy was set to magnitude $t\cdot s\cdot Max_{1}$ and the second to $t(1-s)Max_{2}$, where $Max_{1}$ and $Max_{2}$ correspond to the maximal values for each pair of mixed strategies, t ranges from 0 to 1, and factors s and (1−s) represent relative contributions of the first and second strategies respectively. We examined 11 discrete distributions for each pair $\left(s=0,0.1,\ldots ,1\right)$. For every combination ratio, we simulated the system across a full sweep of parameter t, propulsion forces and axial shifts to identify the specific configuration that yielded the maximum stable curvature for each speed.

**Figure 8.**
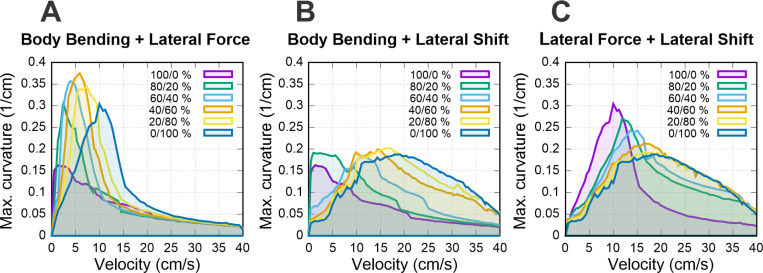
Maximum achievable curvature using combined turning strategies. Each curve represents the peak curvature achievable for a given mixed pair turning strategy for the corresponding velocity with relative contributions indicated in the legend (see text for details). For each mixed pair, propulsion force and axial shift were swept to find the stability boundary.

Our analysis shown in Fig. 8 reveals that combining strategies effectively “fills in the gaps” in performance observed when strategies are used in isolation. While single strategies are dominant in specific speed ranges, their efficacy drops off in the transition zones. The hybrid strategies maintain high curvature in these intermediate zones, creating a continuous envelope of maximum maneuverability across the velocity spectrum.

The results demonstrate that the combination of multiple strategies via simultaneous activation of different asymmetries allows for sharper turns in transition zones where single-strategy performance typically degrades. This effect is most visible in Fig. 8A, where combining body bending and lateral force produces a result that stands out from the other pairings. In this specific case, the two strategies work together to reach substantially higher turning curvatures than what either method can achieve on its own, pushing the model’s maneuverability to a new peak level at low speed.

In contrast, Fig. 8B shows that combining body bending and lateral shift does not make the turns any sharper (i.e increase maximal curvature). Instead, this pairing helps the model produce higher curvatures over a wider range of speeds. Similarly, Fig. 8C shows that the interaction between lateral force and lateral shift mostly serves to bridge the gaps between the two strategies. While this combination helps the model transition smoothly between different modes and fills in performance “valleys”, it does not result in higher turning sharpness than the individual limits established by those strategies.

### Strategy-specific remodeling of limb coordination

**Figure 9.**
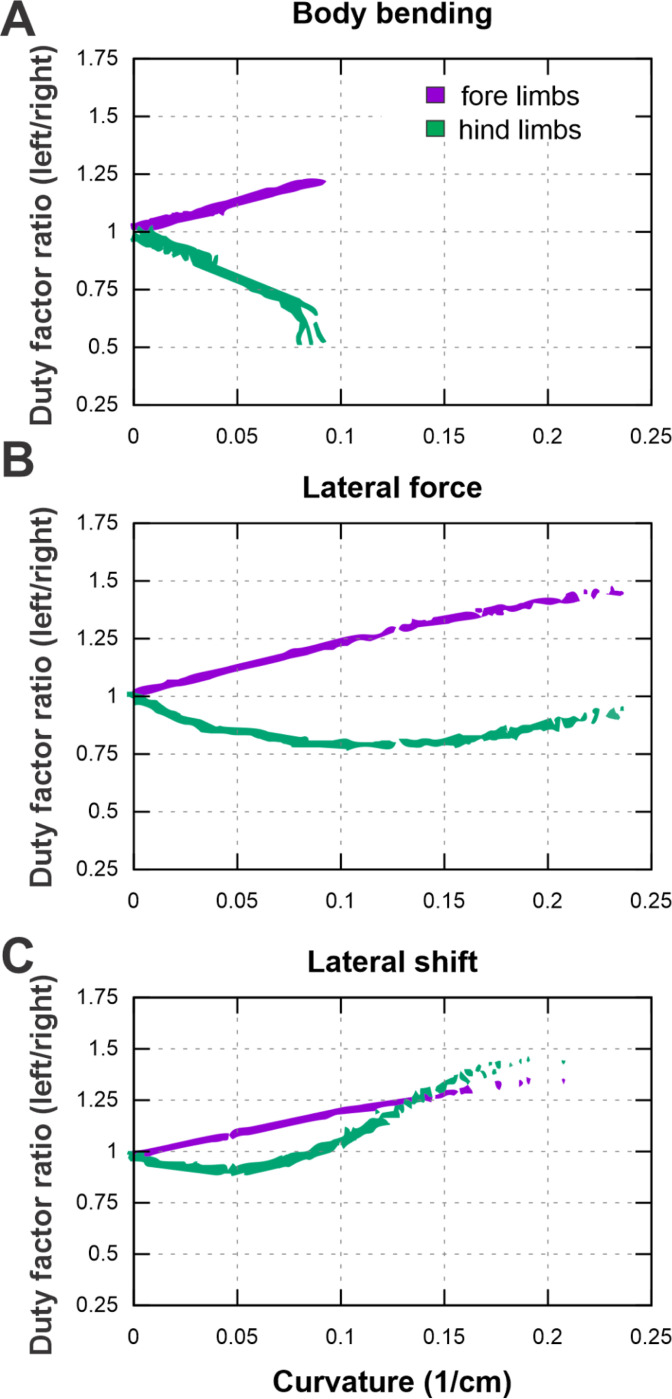
Plots of duty factor ratios (DFR) for forelimbs and hindlimbs as a function of curvature for each turning strategy: body bending (**A**), lateral force (**B**), and lateral shift **(C)**. Forelimbs exhibit a consistent steering role while hindlimbs serve to make strategy-specific propulsion adjustments.

The analysis of limb coordination reveals that turning requires a distinct reorganization of gait timing, quantified here by the ratio of duty factors between inner and outer limbs. Figure 9 shows that while forelimb asymmetry follows a consistent pattern across all strategies, hindlimb coordination is highly strategy-dependent.

Across all three turning strategies—body bending, lateral force, and lateral shift—the forelimbs exhibit a progressive, linear increase in duty factor asymmetry as the turn becomes sharper. This indicates that the inner forelimb consistently spends a longer proportion of the gait cycle in contact with the ground relative to the outer forelimb. In contrast, the hindlimbs display divergent behaviors. In the body bending strategy, hindlimb asymmetry develops in the opposite direction, with the inner limb duty factor decreasing relative to the outer limb as curvature increases. The lateral force strategy shows a non-monotonic pattern: hindlimb asymmetry initially tracks with body bending at low curvatures but reverses trend as the turn tightens. Most notably, the lateral shift strategy produces a sharp, distinct reversal; while it initially resembles the other strategies, at higher curvatures the hindlimb asymmetry spikes dramatically, eventually exceeding the magnitude of asymmetry observed in the forelimbs.

**Figure 10.**
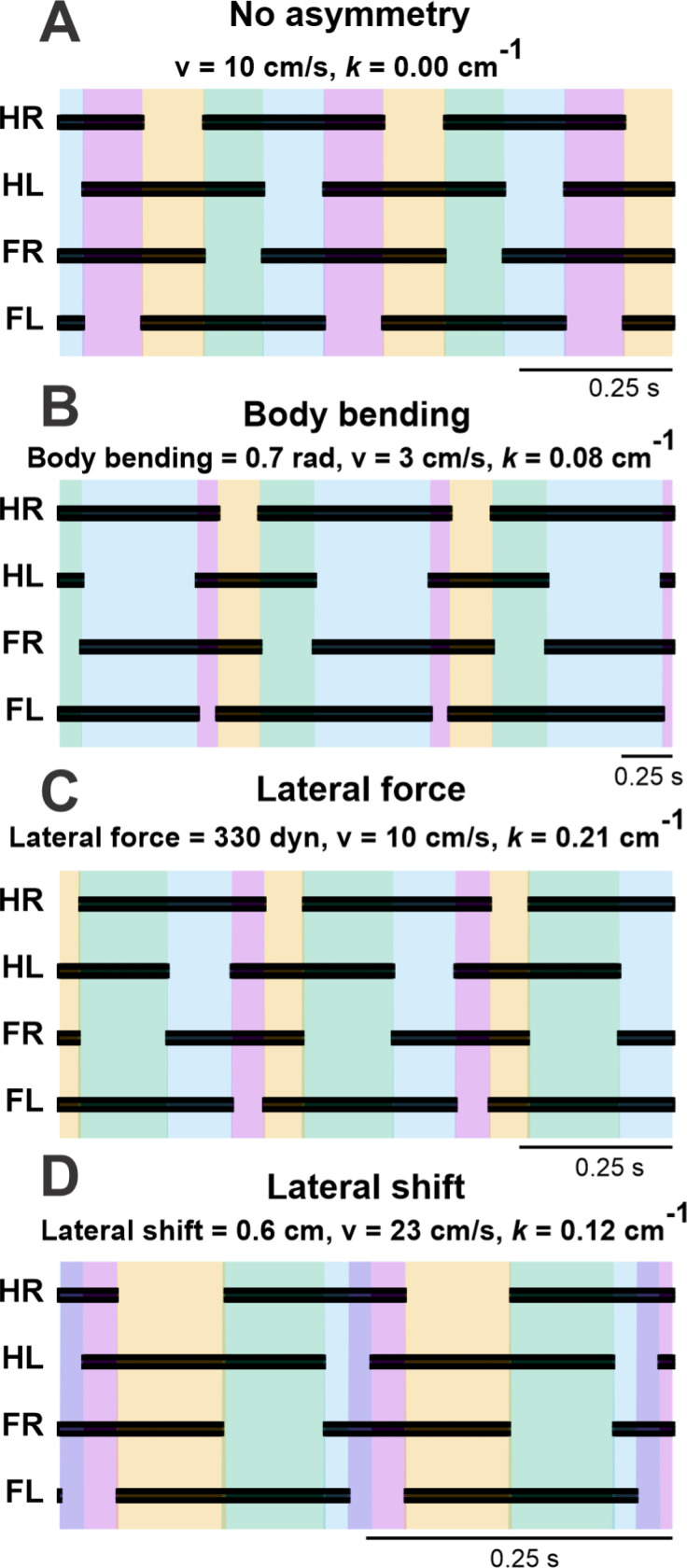
Footfall diagrams for each limb during a (left) turn: hind right (HR), hind left (HL), fore right (FR), and fore left (FL) limbs during straight walking (**A**) and during turns using body bending (**B**), lateral force (**C**) and lateral shift (**D**) strategies, showing temporal sequences of ground contact (black) and swing phases shown by color.

These shifts in timing are visually confirmed by the footfall diagrams in Fig. 10, which depict the temporal sequence of ground contacts. A unique feature emerges specifically within the lateral shift strategy: the synchronization of swing phases on the inner side of the turn. Unlike the baseline gait or other turning strategies where limb cycling remains distinct, the lateral shift strategy at high curvatures leads to a substantial overlap between the swing phases of the inner forelimb and inner hindlimb. This synchronization creates a specific window in the gait cycle where both inner limbs are simultaneously off the ground, leaving the body supported exclusively by the outer limbs.

## Discussion

### Velocity-dependent orchestration of turning strategies

Our results demonstrate that quadrupedal turning is not a monolithic behavior but a dynamic process where strategy selection is fundamentally governed by locomotor velocity. Consistent with observations in biological quadrupeds (Walter, 2003; Gruntman et al., 2007) and bio-inspired robotic models (Wei et al., 2018), we found that no single turning mechanism is optimal across the entire speed range. Instead, the “optimal” strategy shifts from kinematic reconfiguration at low speeds to dynamic force management at high speeds.

At low velocities, the Body Bending strategy dominates by leveraging axial flexibility to reorient the heading direction. This aligns with behavioral studies in rodents showing that spinal curvature is the primary determinant of turning radius during slow exploration (Pycock, 1980). However, as velocity increases, the centrifugal forces associated with sharp turns overwhelm the static stability provided by bending alone. Our model indicates that a transition to the Lateral Force strategy is required. This strategy uses forelimb-driven torque to actively steer the body, effectively “muscling” the system through the trajectory when momentum precludes simple geometric reorientation. Finally, at the highest velocities, the Lateral Shift strategy becomes essential. By widening the base of support on the outside of the turn, this strategy addresses the critical failure mode of high-speed turning: roll-over instability (Lee et al., 2020). This suggests that the quadrupedal nervous system likely prioritizes stability over trajectory sharpness as speed increases, transitioning from steering-focused strategies to stability-focused strategies.

### Mechanistic role of axial shift: coupling geometry and timing

A critical finding of this study is the stabilizing role of the stride control. In our model, this control was achieved by shifting the paw backward as the limb transitions from swing to stance. While often overlooked in simplified models, our results show that shifting the paws posteriorly at touchdown serves a dual biomechanical purpose. Geometrically, it re-centers the support polygon relative to the center of mass (COM), ensuring the COM remains within stable boundaries despite the destabilizing effects of the turn. Temporally, this shift forces a shortening of the effective stride length, which intrinsically increases stepping frequency (Full & Koditschek, 1999).

This frequency modulation reduces the duration of the unstable two-limb support phases, thereby enhancing the dynamic stability margin. However, this comes at a potential metabolic cost due to the higher cadence required. This trade-off implies that the degree of axial shift is likely a tightly regulated parameter in the CNS, tuned to balance safety against metabolic efficiency depending on the urgency and speed of the maneuver.

### Synergy and robustness in multi-modal control

The interaction maps (Fig. 8) suggest that the central nervous system likely employs a “mixed-mode” control strategy rather than switching between independent mechanisms. Notably, the simultaneous activation of Body Bending and Lateral Force (Fig. 8A) produces a synergistic effect, yielding maximal turning curvatures that exceed the limits of either strategy in isolation. This additive performance implies that these two mechanisms are mechanically orthogonal and complementary: Body Bending establishes the geometric arc of the turn by reorienting the body axis, while Lateral Force provides the necessary centripetal acceleration to steer the center of mass along that arc. By combining these distinct mechanical advantages, the system achieves a level of maneuverability that neither strategy can support alone.

However, our current computational analysis was limited to the simultaneous implementation of combined strategies. In contrast, biological quadrupeds, including rodents, often utilize sequential implementations of different strategies or modulate speed to leverage specific speed-dependent mechanisms. For instance, animals may initially reduce locomotor speed—or break movement entirely—to execute an abrupt, but stable, turn using body bending before accelerating again (Pycock, 1980; Walter, 2003; Gruntman et al., 2007; Usseglio et al., 2020). Such complex, temporally distributed turning behaviors represent a higher order of control than the simultaneous combinations modeled here and will be the focus of our future investigations.

### Gait remodeling and the “virtual bank”

The divergence in duty factors (Fig. 9) and the emergence of overlapping swing phases (Fig. 10) in the Lateral Shift strategy point to a sophisticated remodeling of the gait cycle. As the turn tightens, the inner limbs must drastically alter their timing to maintain the overall rhythm despite traveling a shorter path length.

Most notably, the synchronization of inner fore- and hindlimb swing phases creates a transient period where the body is supported exclusively by the outer limbs. Mechanistically, this unloading of the inner side allows gravity to accelerate the COM into the turn, functionally mimicking the “banking” seen in motorcycle dynamics or bipedal running (Jindrich & Qiao, 2009). By momentarily yielding support on the inner side, the animal utilizes gravitational torque to counteract centrifugal force. This suggests that the “instability” of overlapping swings is not a breakdown of the gait, but a controlled maneuver to facilitate high-speed turning dynamics (Usseglio et al., 2020).

### Implications for neural control and robotics

Collectively, these findings support a modular architecture for spinal locomotor control, where distinct neural populations may govern axial flexion, limb loading, and foot placement (Drew et al., 2004). The velocity-dependent efficacy of these strategies suggests that descending commands do not simply specify a “turn” but rather gate and gain-modulate these spinal modules based on sensory feedback regarding speed and stability.

For robotics, this study highlights the limitations of rigid or single-strategy steering controllers. To achieve the agility of biological quadrupeds, robotic platforms must move beyond fixed turning kinematics and implement adaptive, speed-dependent strategy switching (Hattori et al., 2025). Specifically, integrating an active spine and variable limb placement policies (analogous to the Lateral Shift) is critical for expanding the stable operating envelope of legged robots in complex, high-speed environments.

## Conclusion

This study successfully extends the previous model of quadrupedal locomotion (Molkov et al., 2024) to investigate turning strategies in mice. By introducing body bending, lateral force, and lateral shift, we demonstrated that each strategy is optimized for specific speed ranges within the walking gait, suggesting a sophisticated control policy where animals select strategies based on locomotion speed. The forelimbs consistently steer turns, while hindlimbs adaptively adjust propulsion and stability, highlighting the complex interplay between neural control and biomechanical asymmetries. These findings provide a robust framework for understanding adaptive locomotion, with implications for advancing biological research and designing agile robotic systems capable of navigating complex environments (Lee et al., 2020).

## Methods

### Overview

Our foundational approach utilized a comprehensive neuromechanical model originally developed by (Molkov et al., 2024). This model served as the core basis for our simulations and subsequent analyses regarding turning strategies. The model’s key components include representations of the central pattern generators (CPGs) for locomotion, basic biomechanics, and a sensory feedback control, allowing for a dynamic and integrated simulation of locomotor and steering behaviors.

The CPG was simplified as a state machine with each Rhythm Generator (RG) existing in one of two possible states at any given time, corresponding to the limb’s stance and swing phases. The body was modeled as a rigid rod (Fig. 1) confined to the horizontal plane at a constant height. This mechanical system consequently possesses three degrees of freedom: two coordinates for the center of mass (COM) in the horizontal plane and the rod’s orientation.

Body movement within our model arises from propulsive forces generated by limbs in the stance phase. We assume these forces are equal in magnitude and aligned with the paw’s displacement during stance in the body’s coordinate system. During the swing phase, a limb does not contribute to propulsion.

Limb loading is defined as the vertical component of the ground reaction force, calculated based on the COM coordinates relative to the positions of the limbs on the ground. For periods with only two limbs on the ground, the body weight cannot be fully supported, so we incorporated the corresponding horizontal components of the inverted pendulum forces into the equations of motion.

Transitions from stance to swing for each limb occur when either the limb’s loading becomes zero or negative, or the limb’s extension surpasses its limit. Swing-to-stance transitions occur based on a feedback control mechanism for swing duration based on potential loss of balance which is defined as inability of the limbs to fully support the body.

A detailed description of each model component follows below.

### Model of the CPG

We implement the CPG as a set of four rhythm generators (RGs), each controlling one limb. Each RG operates in a state machine regime, so that at each moment in time it can be in one of two states: swing or stance. During stance, the end-effector of the limb (paw), which is controlled by the corresponding RG, is assumed to be on the ground hence providing support for the body. During swing, the limbs instantaneously move to their target position relative to the body and await touchdown.

### Model of the body and limbs

For the mouse’s body, we define a rigid frame with length L and width 2h (Fig. 1). The plane of this frame, also referred to as the *horizontal plane,* is parallel to the ground, and therefore the normal direction to this plane is the *vertical direction*. In terms of mass distribution, the body is considered as a uniform rigid rod of length L with the center of mass (COM) at the midpoint of the body. The distance from the horizontal plane to the ground is defined to be the height H of the mouse’s COM.

The body weight is supported by the limbs that are in stance. The initial positions of the paws in stance correspond to the ground projections of the front of the body’s frame for the forelimbs and of its center (crossing the COM) for the hind limbs (Fig. 1). The initial stance positions of the left and right limbs are displaced from the body’s centerline by distance h to the left and to the right. These initial points serve as target positions in the horizontal plane for the corresponding paws during swing. The maximal possible limb displacement in the horizontal plane from its initial position in the coordinate system associated with the body (maximal limb displacement) is equal to D.

### Equations of motion

Let m be the mass of the mouse and g be the gravitational acceleration. The external forces considered are the gravity force, friction force, and the ground reaction forces. We decompose the forces (where applicable) into components parallel (horizontal) and perpendicular (vertical) to the horizontal plane. Unless otherwise stated, we indicate **vectors by bold letters** and their *magnitudes* using the same notations in an *italic font*. In the following subsections, we decompose the ground reaction forces into their components in the horizontal plane (as those relevant for planar movements of the body) and their vertical components as those providing body weight support.

**Figure 11.**
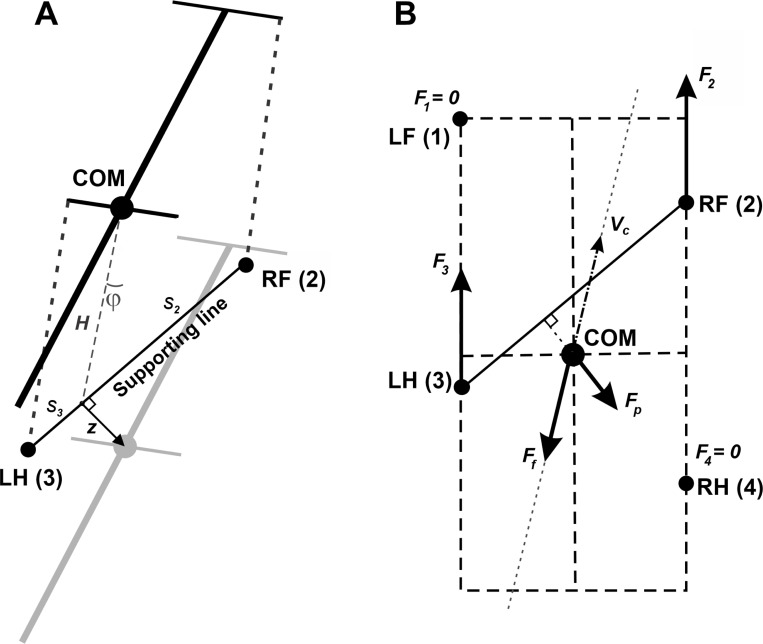
A. Inverted pendulum dynamics during two-leg support. During two-leg support phases, the body weight cannot be fully supported by the limbs on the ground. In this case, we describe the effect of the gravitational force on the horizontal center of mass (COM) movement by the linearized inverted pendulum model. In the example shown, the right fore- (RF (2)) and left hind- (LH (3)) limbs are on the ground providing support, while the left fore- and right hindlimbs (not shown) are in swing. Since the COM is displaced from the supporting line (z is the COM displacement vector in the horizontal plane, φ is the corresponding angle between the projection of the COM onto the line of support and the vertical), the gravitational force creates a rolling torque about this line. We approximate this torque as an equivalent horizontal force pushing the COM in the direction perpendicular to the supporting line (along z). See text for details. **B. A force diagram in the horizontal plane** for the same body configuration as shown in panel A. LF and RH limbs are in swing and do not create propulsive forces. LH and RF push forward with forces $F_{2}$ and $F_{3}$ equal in magnitude and directed along the body axis. The kinetic friction force $F_{f}$ is applied at the COM and directed against the COM velocity $V_{c}$. The inverted pendulum force $F_{p}$ is orthogonal to the line of support connecting LH and RF and pulling the COM away from the line of support. $F_{p}=0$ in case there are more than two limbs on the ground. This sketch is for illustrative purposes only. While it depicts the general concept, it does not reflect the exact proportions or relative force magnitudes of a real mouse. Reproduced from (Molkov et al., 2024).

Let $F_{i}$ denote the component of ground reaction forces in the horizontal plane (2D vector) for limb i (1 for the left fore (LF), 2 for the right fore (RF), 3 for the left hind (LH), and 4 for the right hind (RH) limbs). Specifically, we assume that every paw touching the ground creates a propulsion force $F_{i}$ with magnitude $F_{0}$ (same for all limbs on the ground) directed along the paw’s displacement from its initial position during stance in the body’s coordinate system (see Fig. 11B for an illustration). $F_{0}$ is the main parameter of the model affecting the locomotor speed. Hereinafter, we refer to this parameter as *propulsion force* and use it as one of the control parameters in all our simulations.

During locomotion, energy losses are associated with various factors many of which are not explicitly represented in our simplified model. To account for energy dissipation, we introduce an equivalent viscous friction force that linearly depends on the velocity, i.e., $F_{f}=\lambda v_{c}$, where $v_{c}$ is the COM velocity vector in the horizontal plane and λ is the coefficient of kinetic friction (see Table 1 for parameter values).

When only two limbs support the body, as shown in Fig. 11A, the COM movement in the horizontal plane is affected by gravity. We connect the positions of these two limbs by a supporting line segment. Let z be the distance vector from the supporting line segment to the COM projection on the ground and let be the angle of inclination (Fig. 11A). The model can be seen as an inverted pendulum pivoted at the supporting line segment governed by the (linearized) equation $z^{\prime \prime }=gz/H$, where prime (′) denotes the derivative with respect to time. This acceleration multiplied by the mass m represents an *inverted pendulum force*, which we denote here by $F_{p}=mgz/H$. If more than two limbs are on the ground, the COM is always inside of the supporting triangle or quadrangle (because otherwise at least one of the limbs would have a negative load and therefore could not be on the ground), $F_{p}=0$.

By Second Newton’s Law, the velocity $v_{c}$ obeys the following equation (Fig. 11B):

(1) $$mv_{c}^{\prime }=F_{1}+F_{2}+F_{3}+F_{4}-\lambda v_{c}+F_{p}$$

Since the propulsion forces are displaced from the axis of the body, they will contribute to the yaw torque leading to the rotation of the body around the COM in a horizontal plane. The moment of inertia I of a uniform rod of length L about the COM is $I=mL^{2}/12$.

The gravity force is applied at the COM, thus creating zero torque. Let $r_{c}$ be the position vector of the COM in the horizontal plane, and $r_{i}$ be the position vector of paw i on the ground. Then, the yaw torque that each propulsion force creates is $M_{i}=F_{i}\times \left(r_{i}-r_{c}\right)$. The friction torque $M_{f}$ of the rod can be calculated as $M_{f}=\lambda l\omega /m$, where ω is the angular velocity of the body rotation about the COM. By Second Newton’s Law in angular form, the following differential equation describes the rotation of the body about the COM in the horizontal plane:

(2) $$I\omega^{\prime }=M_{1}+M_{2}+M_{3}+M_{4}-M_{f}$$

### Weight bearing forces

To calculate the vertical components of the ground reaction forces $\left(G_{i}\right)$ in each limb that we also refer to as weight-bearing or supporting forces, we consider different possible situations depending on the number of limbs in stance.

#### Support by more than two limbs:

We assume that the body frame always remains in the horizontal plane and, therefore, is not pitching or rolling. Therefore, the total torque created by the supporting forces should be balanced, i.e.


(3) $$\sum_{i}G_{i}\times \left(r_{i}-r_{c}\right)=0.$$

In addition, for the body to remain in the horizontal plane (not move in the vertical direction), the total supporting force should be equal in magnitude to the gravitational force:

(4) $$\sum_{i}G_{i}=mg$$

Finally, ground reaction forces are zero for all limbs in swing.

Note that Eq. (3) contains two equations as vector products in the sum have two non-trivial components. So, Eqs. (3) and (4) define a linear system of three equations for the supporting forces. This system has a unique solution in the case of three-leg support (provided that the three paws are not positioned on a single straight line). In the case of four-leg support an additional constraint is necessary as different weight distributions that satisfy Eqs. (3) and (4) are possible. For four-leg support, we assume that the total load is distributed as evenly as possible. Particularly, we find a solution of Eqs. (3) and (4) that minimizes $\Sigma_{\text{i}}\;G_{i}^{2}$ using the method of Lagrange multipliers.

For a limb to stay on the ground, the upward component of its ground reaction force must be positive. Geometrically, the system of Eqs. (3) and (4) has positive solutions only if the COM is located inside the triangle (or quadrangle) formed by the limbs in contact with the ground. Whenever the COM falls on the edges of this triangle (quadrangle), the supporting force (load) in the limb opposite to that edge reduces to zero, indicating that the limb needs to be lifted.

#### Support by two limbs:

In case of only two limbs being on the ground, the system of Eqs. (3) and (4) does not generally have solutions meaning that the body weight cannot be fully supported (unless the COM is precisely above the line of support, see Fig. 11). Here we take the case of the diagonal limbs in stance as an example. Consider the left hind- (i = 3) and right fore- (i=2) limbs are supporting and the other two limbs (i = 1 and 4) are in swing (as shown in Fig. 11), that is, $G_{1}=G_{4}=0$. The movement of this frame in the direction perpendicular to the supporting line will follow the inverted pendulum dynamics as illustrated in Fig. 11. Let $v_{p}$ be the COM velocity component in the direction perpendicular to the supporting line. By taking the centripetal force into account, for the vertical component of the ground reaction force we have:

(5) $$G_{2}+G_{3}=mg\;\text{cosφ}-mv_{p}^{2}/H$$

To find the load distribution over limbs 2 and 3 we assume that there is no pitch in the direction of the supporting line, so the torque created by $G_{2}$ and $G_{3}$ about the projection of the COM onto the line of support must be zero. Specifically,

(6) $$G_{2}\;s_{2}=G_{3}\;s_{3},$$

where $s_{2}$ and $s_{3}$ are the segments of the line of support between the projection of the COM on it and the positions of paws 2 and 3, respectively (see Fig. 11). After solving the linear system (5), (6) we find ${\text{G}}_{2}$ and ${\text{G}}_{3}$ for the case that the body is supported by limbs 2 and 3 only. Similarly, we find the vertical components of the ground reaction forces (also referred to as weight-bearing forces and limb load) for any other pair of supporting limbs during 2-leg support phases.

### Stance-to-swing transition

The transition from stance to swing (lift off) for each limb is governed by local proprioceptive feedback. Modeled after mechanisms observed in biological quadrupedal locomotion (Ekeberg & Pearson, 2005; Molkov et al., 2024), the rhythm generator for a specific limb switches from the stance state to the swing state if either of the following two conditions is met:

#### Limb Unloading (Force-dependent feedback):

The limb is lifted if its load—defined as the vertical component of the ground reaction force—drops to zero or becomes negative ($G_{i}<0$). This rule ensures that a limb does not lift while it is essential for supporting the body’s weight, mimicking the inhibitory feedback from ankle extensor load receptors (e.g., Golgi tendon organs) that prevents swing initiation during substantial weight bearing.

#### Limb Extension (Geometry-dependent feedback):

The limb is lifted if it reaches its maximum posterior displacement limit. The model monitors the displacement of the paw $\left(\left|\text{Δ}r_{i}\right|\right)$ relative to its initial touchdown position in the body’s coordinate system. If this displacement exceeds a predefined maximum limb displacement $\left(D\right)$, the limb is forced to lift $\left(\left|\text{Δ}r_{i}\right|>D\right)$. This mimics the signal from hip flexor stretch receptors which triggers the flexor burst to initiate swing when the limb is fully extended.

### Swing-to-stance transition

The transition from swing to stance (touchdown) is governed by the need to maintain static stability. This phase transition is triggered by a “loss of balance” mechanism, which ensures that a swinging limb is grounded immediately if the body’s stability is threatened.

In this model, loss of balance is defined as a negative rate of change of the total load (total vertical ground reaction force, $G_{tot}=\Sigma_{i}\;G_{i}$). When the total load begins to decrease (i.e., $G_{tot}^{\prime }<0$), indicating that the current support polygon is insufficient to maintain the body’s height, the swing phase must be terminated.

If multiple limbs are currently in the swing phase when this condition is met, the system selects the limb that has been swinging for the longest time to transition to stance. This selection criterion prioritizes the limb that is furthest along in its swing trajectory, ensuring a more natural and efficient recovery of stability.

### Axial (sagittal) shift

To enable precise control over stride length and stepping frequency during locomotion, we incorporated an axial shift mechanism into the model. This strategy involves a controlled rearward displacement of the target paw position along the body’s longitudinal axis at the transition from swing to stance. Specifically, upon initiating stance, the initial paw placement is shifted backward by a variable amount (a control parameter we refer to as *axial shift*) relative to its default unperturbed position in the body coordinate system. This axial shift effectively shortens the stride length without altering the propulsion force magnitude, thereby allowing modulation of gait parameters such as step frequency and overall speed. By reducing the distance the body must travel over a fixed paw during stance, the mechanism increases the frequency of phase transitions.

In implementing this mechanism, the maximal limb displacement parameter D (see Table 1) is reduced for the forelimbs by the same amount as the axial shift applied to both fore- and hindlimbs. This adjustment is necessary to maintain equivalent duty factors—the ratio of stance duration to total stride cycle—between the forelimbs and hindlimbs. Without this compensation, the differing axial attachment points of the limbs (forelimbs at the front of the body frame and hindlimbs at the center, aligned with the COM) would result in asymmetric effective displacements during stance. The forelimbs, positioned further anteriorly, would inherently experience longer relative displacements under the same propulsion conditions, leading to prolonged stance phases and higher duty factors compared to the hindlimbs. Such mismatch could disrupt gait symmetry, destabilize postural control, and impair the coordination required for stable forward locomotion. By reducing D for the forelimbs, the model ensures balanced duty factors across all limbs, preserving the integrity of symmetrical gaits while allowing the axial shift to serve as an effective stride control tool. This approach aligns with observed biological adaptations in quadrupeds, where neural feedback modulates limb placement to optimize energy efficiency and stability across varying speeds (Ijspeert & Daley, 2023).

### Implementation of asymmetries for turning

To investigate turning behaviors, we extended the baseline symmetrical model by implementing three distinct biomechanical asymmetries. These strategies introduce controlled deviations from bilateral symmetry to generate the rotational moments required for changing direction.

### Body Bending (geometric asymmetry)

This strategy mimics the spinal flexion observed in biological quadrupeds during turning. In the baseline model, the body is a single rigid rod. To implement bending, we introduce an angular deviation, δ (ranging from 0 to 1 radians), between the front and rear segments. The body is effectively treated as two segments where the front segment is rotated by δ/2 counter-clockwise (clockwise) and the rear segment is rotated by δ/2 clockwise (counter-clockwise) for a left (right) turn. This geometric transformation reorients the limb attachment points in the body coordinate system. Consequently, the swing targets and the resulting support polygon are shifted, generating a yaw torque through the redistribution of ground reaction forces without altering the magnitude of propulsion.

### Lateral Force (dynamic asymmetry)

This strategy simulates the active generation of lateral ground reaction forces, primarily used for steering. We applied a lateral force vector perpendicular to the body axis, directed inward toward the center of the turn (e.g., leftward for a left turn). This force is applied exclusively to the forelimbs to mimic their steering role, while the hindlimbs receive zero lateral force component. The addition of this perpendicular component modifies the horizontal equations of motion by introducing a centripetal force in Eq. (1) and an asymmetric yaw torque in Eq. (2) thereby driving trajectory curving and rotation of the body.

### Lateral Shift (kinematic asymmetry)

This kinematic asymmetry involves offsetting the mediolateral placement of the limbs relative to the body’s midline specifically at the moment of the swing-to-stance transition. In the baseline model, limbs are displaced by a fixed half-width h from the center (see Table 1). For a turn, we introduce a lateral offset parameter s that modifies the target touchdown coordinates. Specifically, inner limbs (e.g., left) are shifted inward, reducing their distance from the midline to h−s, while outer limbs (e.g., right) are shifted outward, increasing their distance from the midline to h+s This shift alters the geometry of the support polygon and the direction of the propulsion forces. Because propulsion is directed along the paw displacement vector, shifting the starting position naturally introduces a lateral component to the propulsion force vector during stance.

### Calculation of turning curvature

To quantify turning performance, we calculated the signed curvature of the locomotor trajectory using Menger curvature. Menger curvature is a geometric measure defined for a set of three distinct points as the reciprocal of the radius of the unique circumcircle passing through them $\left(\varkappa =1/R\right)$. This approach allows for a precise, discrete approximation of curvature without requiring a continuous path derivative.

In our analysis, we calculated this curvature for each stride using the COM coordinates recorded at three consecutive touchdowns of the left forelimb. This method effectively captures the sustained turning radius of the model during stable locomotion.

The reported curvature for each simulation corresponds to the stabilized value of ϰ once the gait reaches a steady state. Positive values of ϰ indicate leftward turns, while negative values indicate rightward turns; the magnitude reflects the sharpness of the turn, with higher values corresponding to tighter radii $\left(1/\varkappa \right)$.

## Figures and Tables

**Table 1.** Model parameters.

Name	Notation	Value
Length of the animal	L	10 cm
COM height	H	3 cm
Half width	h	1 cm
Maximal limb displacement	D	5 cm
Mass	m	25 g
Friction coefficient	λ	100 g/s
Gravitational acceleration	g	1000 cm/s^2^
